# The value of sonication in the differential diagnosis of septic and aseptic femoral and tibial shaft nonunion in comparison to conventional tissue culture and histopathology: a prospective multicenter clinical study

**DOI:** 10.1186/s10195-023-00708-4

**Published:** 2023-06-12

**Authors:** Katharina Trenkwalder, Sandra Erichsen, Ferdinand Weisemann, Peter Augat, Matthias Militz, Christian von Rüden, Tobias Hentschel, SAND Research Group, Heiko Baumgartner, Marie Reumann, Georg Reiter, Holger Freischmidt, Matthias Kemmerer, Steffen Langwald, John Hanke, Martin Glombitza, Eva Steinhausen, Ulf-Joachim Gerlach, Nikolai Spranger, Dirk Stengel, Simon Hackl

**Affiliations:** 1 Institute for Biomechanics, BG Unfallklinik Murnau, Professor-Küntscher-Str. 8, 82418 Murnau am Staffelsee, Germany; 2 Institute for Biomechanics, Paracelsus Medical University, Strubergasse 21, 5020 Salzburg, Austria; 3 Department of Trauma Surgery, BG Unfallklinik Murnau, Professor-Küntscher-Str. 8, 82418 Murnau am Staffelsee, Germany

**Keywords:** Septic nonunion, Aseptic nonunion, Fracture-related infection, Low-grade infection, Sonication, Membrane filtration, Tissue culture, Histopathology

## Abstract

**Background:**

Septic and aseptic nonunion require different therapeutic strategies. However, differential diagnosis is challenging, as low-grade infections and biofilm-bound bacteria often remain undetected. Therefore, the examination of biofilm on implants by sonication and the evaluation of its value for differentiating between femoral or tibial shaft septic and aseptic nonunion in comparison to tissue culture and histopathology was the focus of this study.

**Materials and methods:**

Osteosynthesis material for sonication and tissue samples for long-term culture and histopathologic examination from 53 patients with aseptic nonunion, 42 with septic nonunion and 32 with regular healed fractures were obtained during surgery. Sonication fluid was concentrated by membrane filtration and colony-forming units (CFU) were quantified after aerobic and anaerobic incubation. CFU cut-off values for differentiating between septic and aseptic nonunion or regular healers were determined by receiver operating characteristic analysis. The performances of the different diagnostic methods were calculated using cross-tabulation.

**Results:**

The cut-off value for differentiating between septic and aseptic nonunion was ≥ 13.6 CFU/10 ml sonication fluid. With a sensitivity of 52% and a specificity of 93%, the diagnostic performance of membrane filtration was lower than that of tissue culture (69%, 96%) but higher than that of histopathology (14%, 87%). Considering two criteria for infection diagnosis, the sensitivity was similar for one tissue culture with the same pathogen in broth-cultured sonication fluid and two positive tissue cultures (55%). The combination of tissue culture and membrane-filtrated sonication fluid had a sensitivity of 50%, which increased up to 62% when using a lower CFU cut-off determined from regular healers. Furthermore, membrane filtration demonstrated a significantly higher polymicrobial detection rate compared to tissue culture and sonication fluid broth culture.

**Conclusions:**

Our findings support a multimodal approach for the differential diagnosis of nonunion, with sonication demonstrating substantial usefulness.

*Level of Evidence*: Level 2

*Trial registration* DRKS00014657 (date of registration: 2018/04/26)

## Introduction

One essential criterion for the treatment of fracture nonunion is the presence or absence of bacterial infection. However, differentiating between septic and aseptic nonunion is challenging, especially when dealing with low-grade infections which are difficult to distinguish from aseptic nonunion [1, 2]. Low-grade infections are often caused by biofilm-forming bacteria, which may remain undetected in tissue cultures (TCs) and erroneously lead to aseptic diagnoses [2–4].

Biofilm-embedded bacteria on implants can be dislodged by sonication and detected by sonication fluid (SF) culture. This method has become an important element in the diagnosis of prosthetic joint infection (PJI) due to its supposed higher sensitivity compared to TC [5], despite some recent contradictory findings [6–8]. There are few studies focusing on sonication for the diagnosis of fracture-related infection [9–13], with most of the studies including both fracture-related and prosthetic joint infections [9, 11–13]. Thus, the role of sonication in the diagnosis of fracture-related infection (FRI) remains tentative [14]. Based on the lack of scientific evidence for the role of sonication in FRI diagnostics [14], the FRI Consensus Group demanded further studies to establish the role of sonication in FRI [15].

The aim of this study was to evaluate the diagnostic performance of sonication in differentiating between diaphyseal femoral or tibial aseptic and septic nonunion. We hypothesized that differentiation would be possible by the quantification of colony forming units (CFU) in sonication fluid. Furthermore, we hypothesized that sonication would detect a more diverse spectrum of microorganisms compared with tissue culture.

## Patients and methods

This prospective multicenter study investigated the microbiological colonization of femoral and tibial fixation material in nonunion and regular healed fractures. One hundred thirty patients were recruited at eight level I trauma centers in Germany between January 2019 and April 2022. Each study center received an ethics vote from its local responsible ethics committee. Patients with aseptic or septic femoral or tibial shaft nonunion admitted to the hospitals for a revision surgery and patients with femoral or tibial regular healed fractures undergoing implant removal were included in this study. In the latter, the implant was routinely removed after complication-free fracture consolidation, with no clinical or laboratory signs of infection or delayed healing during the fracture healing process. All patients gave their written consent before study inclusion.

### Study population

Ninety-eight patients with nonunion and 32 patients with regular healed fractures aged ≥ 18 years were prospectively identified at the participating study centers and included in the study. The diagnosis of nonunion was based on the patient´s complaints, clinical examination, and mandatory conventional radiographs. Hereby, radiologic signs of nonunion were defined as a lack of osseous bridging in at least three out of four cortices assessed on antero-posterior and lateral views of conventional radiographs [16]. Nonunion was defined as a fracture that does not heal without further surgical intervention [17]. The median time between definitive fracture stabilization and revision surgery was 10.9 months (range: 2.4–48.9). Exclusion criteria were administration of preoperative long-term antibiotics or current antimicrobial therapy and pregnancy. Only patients with complete implant removal or replacement were involved in this investigation.

Septic nonunion was defined according to Metsemakers et al. [18] by confirmatory criteria: presence of a fistula, two of the same pathogens identified in separate TCs, two of the same pathogens in TC and SF broth culture, or the detection of microorganisms in one TC confirmed by histopathological examination. For all patients, microbiological (long-term culture of tissue in broth and direct culture) and histopathological findings from index surgery or any follow-up surgeries within 12 months of study inclusion were considered for diagnosis. Especially in the case of a single culture-positive specimen or histopathology with infection signs only, suggestive criteria (redness, swelling, increased local temperature, increased preoperative CRP, clinical suspicion) were taken into account for the definitive diagnosis. Additionally, the administration of nonunion-related antibiotic therapy as well as the consolidation status of the nonunion 1 year after revision surgery were recorded. Results of the study-related sonication fluid membrane filtration (MF) were not considered as a diagnostic criterion, as the evaluation of this method was subject of the present study.

### Sample collection and analysis

The purpose of this study was to evaluate the diagnostic performance of sonication in differentiating between septic and aseptic nonunion in order to improve and expedite the diagnostic process for early initiation of appropriate treatment. Therefore, we assessed sonication as a diagnostic method in comparison to current conventional diagnostics.

Nonunion revisions and metal removals were performed according to clinical standards. Besides the clinical sampling for microbiology and histopathology (study-center-dependent sampling and analysis with local variations), two additional tissue samples were obtained from the transition zone between healthy bone and nonunion in nonunion patients and from the peri-implant site in regular healers. These samples were directly transferred into prepared vials, one in 9 ml sterile thioglycolate broth with resazurin (bioMérieux) for conventional microbiological examination and the other in 4%-phosphate-buffered formaldehyde solution (AppliChem) for histopathological examination. Osteosynthesis material was removed under sterile conditions according to clinical standards and transferred dry into sterile stand-up bags (Whirl-Pak, Nasco Sampling). All study samples collected in the study surgeries were transported overnight to the Institute for Biomechanics at the BG Unfallklinik Murnau, and were processed on the subsequent day.

Sonication was performed with the entire intramedullary nail or locking plate; screws were included if provided. The removed osteosynthesis material was transferred into a steam-sterilized (121 °C, 20 min) custom-made screw-capped glass tube with space for a complete intramedullary nail. Implants that did not fit into the glass tubes were instead processed in hydrogen-peroxide-sterilized lock’n’lock containers recommended by the ultrasonic bath manufacturer for sonication. The osteosynthesis material was covered with 150 ml sterile Ringer’s solution (Ringer Fresenius, Fresenius Kabi Deutschland GmbH) and shaken by hand for 30 s. Sonication was performed with a frequency of 40 kHz at 100% power setting (corresponds to 200 W_eff_ ultrasonic nominal output; BactoSonic ultrasonic bath, Bandelin electronic GmbH & Co. KG) for 1 min. For long implants that protruded out of the ultrasonic bath, the glass tubes were inverted and sonicated for an additional minute to include the whole implant surface. Sonication was followed by further shaking for 30 s. Five ml of the SF was inoculated into 9 ml thioglycolate broth with resazurin (bioMérieux) and incubated at 37 °C, 5% CO_2_ for 10 days.

**Fig. 1:**
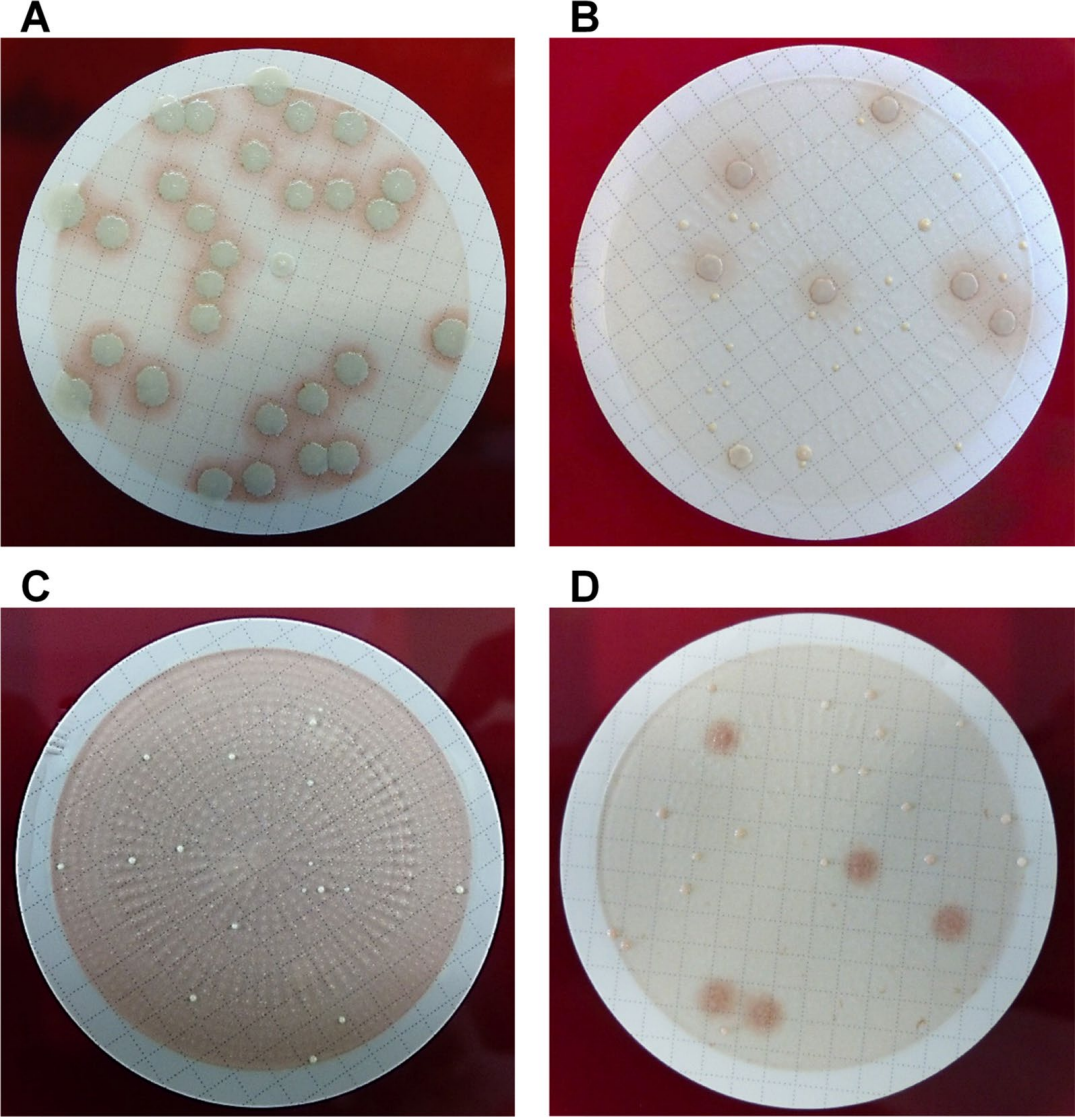
Examples of aerobically (**A**, **B**) and anaerobically (**C**, **D**) incubated filter membranes with bacteria growth. **A** *Staphylococcus capitis*. **B** *Corynebacterium* spp. and* Staphylococcus epidermidis*. **C** *Cutibacterium acnes*. **D** *Cutibacterium* *acnes* and *Staphylococcus epidermidis*

Membrane filtration (MF) was conducted with 80 ml SF in total. For this purpose, SF was pre-filtered through a 70 μm sterile cell strainer (Falcon, Corning, NY, USA) to remove larger particles and to avoid blocking the 0.45-μm-pore-size sterile membrane filters (EZ-Fit, Millipore, Merck KGaA). Four filtrations with 20 ml each were performed. Filter membranes were directly placed onto Columbia agar plates with 5% sheep blood (Oxoid Germany GmbH, Thermo Fisher Scientific). Two agar plates were incubated under aerobic conditions for at least 2 days [median (range): 3 (2–7)], and two under anaerobic conditions for at least 5 days [median (range): 5 (5–7)], with variations due to weekends and holidays. An Anoxomat system (Advanced Instruments, Norwood, USA) was used to generate an anaerobic atmosphere. After incubation, the CFU were quantified on either aerobically or anaerobically incubated filter membranes (whichever yielded higher counts according to Trampuz et al. [19]). For this purpose, photos of the filter membranes were taken (Fig. 1) and the CFU were visually enumerated using the multi-point tool of the ImageJ software (version 1.52f). Only colonies from which at least one other colony of the same pathogen had grown on the selected membrane filters were counted. Individual colonies were considered contaminants and were not assessed. Eight sterile intramedullary nails and plates were transferred into sterile stand-up bags (Whirl–Pak, Nasco Sampling) and tested as negative controls in the same manner.

The study TC samples were incubated at 37 °C, 5% CO_2_ for 14 days and then streaked out on Columbia agar with 5% sheep blood (Oxoid Germany GmbH, Thermo Fisher Scientific). Morphologically distinct colony types were identified by matrix-assisted laser desorption ionization time-of-flight (MALDI-TOF) mass spectrometry to species level (Vitek MS, bioMérieux Vitek Inc.) and tested for antibiotic susceptibility (Vitek 2, bioMérieux Vitek Inc.). The 10-day culture of SF in thioglycolate broth was performed in the same manner. Morphologically distinct colony types grown on membrane filters of MF were isolated on Columbia agar with 5% sheep blood (Oxoid Germany GmbH, Thermo Fisher Scientific) and identified and tested for antibiotic susceptibility analogously to TC and SF broth culture samples.

For histopathology, tissue samples were fixated in formaldehyde and were examined for signs of osteomyelitis after decalcification. Histopathological assessment and classification was performed via H&E staining, Berlin blue and the PAS reaction and with polarization-optical analysis and a semi-quantitative evaluation of osteomyelitis according to the Histopathological Osteomyelitis Evaluation Score (HOES [20]). In brief, patterns of acute and chronic osteomyelitis, which include osseous changes, soft-tissue changes and an inflammatory infiltrate pattern (microabscesses: ≥ 5 neutrophilic granulocytes; lymphocyte/macrophage/plasma cell infiltrate), were assessed and semi-quantitatively graded depending on the size of the section area (non-existent = 0, one-third = 1, two-thirds = 2, entire section area = 3). Numerical evaluations of osteomyelitis criteria were summed and resulted in the written HOES, whereby scores of I to IV indicate the presence of osteomyelitis at different stages (acute, chronically florid, chronic, calmed osteomyelitis) and a score of V implies that there is no indication of osteomyelitis. For our analyses, we only used the HOES to differentiate whether signs of osteomyelitis were present or not.

### Statistics

Intraoperative tissue specimens and explanted osteosynthesis material of 130 patients that met the defined inclusion criteria were sampled and analyzed in this study. Three initially aseptic nonunion patients were excluded from the statistical analyses as their signs of infection first appeared in follow-up surgeries, resulting in an unclear differential diagnosis. Thus, 127 patients were included in further analyses.

Statistical analyses were conducted in SPSS (ver. 26; IBM). Comparisons of mean values between study groups were performed using the Kruskal–Wallis or Mann–Whitney* U* test. To compare qualitative variables, Pearson’s chi-squared test or Fisher’s exact test was applied. A *p* value < 0.05 (two-sided) was considered statistically significant, and Bonferroni correction was performed for multiple testing. CFU cut-off values and sensitivity and specificity for infection diagnosis based on the MF method were calculated with receiver operating characteristic (ROC) curves . Two by two tables were used for to test the performances of the different test methods. Analyses for the single-test methods were performed with the study-related microbiological and histopathological samples only, which were processed in a standardized manner. 95% confidence intervals (CIs) for sensitivity, specificity, positive and negative predictive values (PPV and NPV) were generated via Clinical Calculator 1 (http://vassarstats.net/).

## Results

The study groups—42 patients with septic nonunion, 53 with aseptic nonunion and 32 with regularly healed fractures—significantly differed in age (Table 1). In particular, the regular healer group was significantly younger than the aseptic nonunion group (*p* = 0.006), whereas there was no difference in age between septic and aseptic nonunion (*p* = 1.000) or septic nonunion and regular healers (*p* = 0.072). The distribution of open and closed fractures differed significantly between the groups, as open fractures occurred more frequently in nonunion compared to regular healers (*p* = 0.005). The number of tissue cultures for the clinical diagnosis of infection was larger for septic nonunion (4 ± 2) compared to aseptic nonunion (3 ± 2; *p* = 0.006). There were no significant between-group differences regarding type of osteosynthesis or sonication conditions (Table 2). Revision surgery with implant removal occurred at a median of 11.7 months (range: 2.4–48.9) after definitive stabilization in the septic nonunion group and at a median of 10.1 months (range: 4.1–41.2) in the aseptic nonunion group. In the regular healer group, routine metal removal was performed at a median of 15.1 months (range: 9.5–28.9) after internal fracture fixation.

**Table 1:** Comparison of patient characteristics between the study groups

Characteristics of the patients	Septic nonunion		Aseptic nonunion		Regular healers		*p * value
	*N*	*%*	*N*	*%*	*N*	*%*	
Sex							0.092^a^
Male	34	81	32	60	21	66	
Female	8	19	21	40	11	34	
Age							**0.007**^**b**^
Mean ± SD [years]	45 ± 15		48 ± 15		38 ± 16		
Fracture							**0.003**^**a**^
Closed	20	48	28	53	27	84	
Open	22	52	25	47	5	16	
Bone							0.358^a^
Femur	13	31	24	45	12	38	
Tibia	29	69	29	55	20	63	
Fracture localization							0.155^c^
Proximal shaft third	4	10	4	8	6	19	
Medial shaft third	14	33	29	55	12	38	
Distal shaft third	22	52	17	32	14	44	
Two different shaft thirds	2	5	3	6			

^a^Pearson's chi-square test

^b^Kruskal–Wallis test

^c^Fisher’s exact test

**Table 2:** Comparison of sample characteristics between the study groups

Sample characteristics	Septic nonunion		Aseptic nonunion		Regular healers		*p* value
	*N*	*%*	*N*	*%*	*N*	*%*	
Sonicated osteosynthesis material							0.086^a^
Intramedullary nail (+ screws)	28	67	37	70	29	91	
Plate (+ screws)	12	29	15	28	3	9	
Intramedullary nail + plate (+ screws)	2	5	1	2			
Sonication container							0.128^a^
Glass tube	38	91	47	89	32	100	
Lock'n'lock box	4	10	6	11			

^a^Fisher’s exact test

### Detection of septic nonunion by CFU quantification

**Fig. 2:**
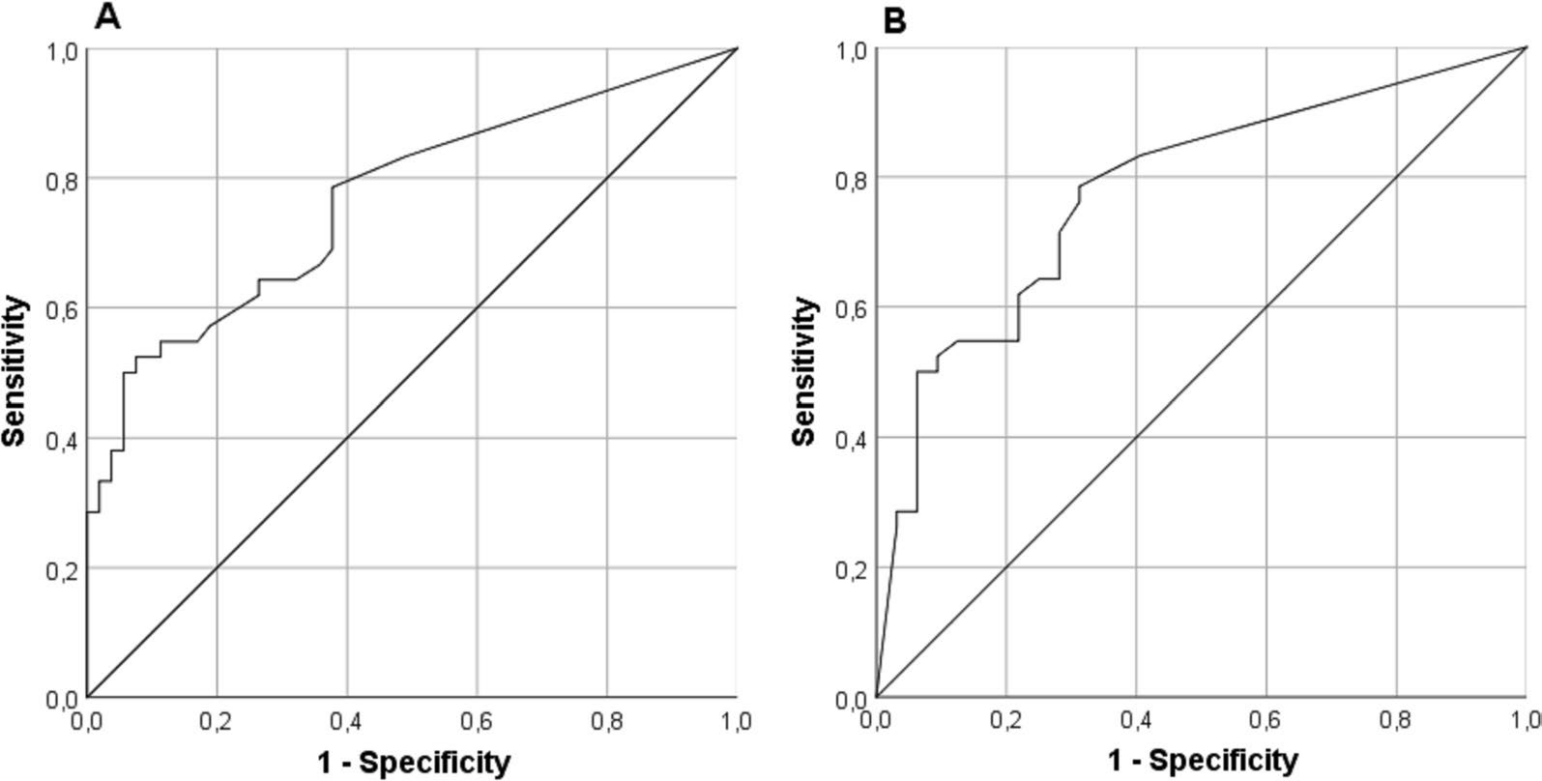
ROC curves for group discrimination based on CFU per ml sonication fluid (SF) determined by the membrane filtration method. **A** Discrimination of aseptic and septic nonunion; AUC = 0.77 (95% CI 0.67–0.87); optimal cut-off value that maximizes the Youden index (0.45) is ≥ 13.6 CFU/10 ml SF. **B** Discrimination of regular healed fractures and septic nonunion; AUC = 0.78 (95% CI 0.67–0.89); optimal cut-off value that maximizes the Youden index (0.47) is ≥ 0.6 CFU/10 ml SF

ROC analyses for the MF method were performed for separation between septic and aseptic nonunion and between septic nonunion and regular healers, respectively. Both ROC curves had an area under the curve of 0.8. The ROC curve analysis for the best cut-off value revealed ≥ 13.6 CFU/10 ml SF for differentiation between aseptic and septic nonunion (high cut-off; Fig. 2A) and ≥ 0.6 CFU/10 ml SF for differentiation between regular healers and septic nonunion (low cut-off; Fig. 2B).

A large spectrum of different bacterial species were detected in all three study groups (Table 3). In septic nonunion, coagulase-negative staphylococci (CoNS) and *Cutibacterium acnes* were the most commonly detected pathogens. In aseptic nonunion and regular healers, the majority of samples were culture negative. Positive SF cultures in aseptic nonunion or regular healers were mainly due to CoNS and *C. acnes*. MF demonstrated a higher detection rate of polymicrobial infections compared to TC and broth-cultured SF (*p* < 0.001). All negative controls for sonication had no bacterial contamination.

**Table 3:** Overview of detected microorganisms in the three study groups

Study group	Culture results	TC		SF broth culture		MF	
		*N*	%	*N*	%	*N*	%
Septic nonunion (*N* = 42)	Negative	13	31	13	31	20	48
	1 species/sample	26	90	26	90	11	50
	2 species/sample	3	10	3	10	7	32
	3 species/sample	–	–	–	–	4	18
	*Cutibacterium acnes*	10	31	3	9	6	16
	CoNS	16	50	24	75	25	68
	*Staphylococcus aureus*	4	13	1	3	1	3
	*Finegoldia magna*	1	3	2	6	1	3
	*Serratia marcescens*	1	3	1	3	1	3
	*Enterococcus faecalis*	–	–	1	3	1	3
	*Corynebacterium* spp.	–	–	–	–	2	5
Aseptic nonunion (*N* = 53)	Negative	51	96	43	81	49	93
	1 species/sample	2	100	10	100	–	–
	2 species/sample	–	–	–	–	3	75
	3 species/sample	–	–	–	–	1	25
	*Cutibacterium acnes*	–	–	4	40	3	33
	CoNS	1	50	5	50	5	56
	*Kocuria varians*	1	50	–	–	–	–
	*Brevibacillus choshinensis*	–	–	1	10	–	–
	*Corynebacterium* spp.	–	–	–	–	1	11
Regular healers (*N* = 32)	Negative	29	91	23	72	29	91
	1 species/sample	2	67	8	89	2	67
	2 species/sample	1	33	1	11	1	33
	*Cutibacterium acnes*	2	50	1	10	2	50
	CoNS	1	25	5	50	1	25
	*Corynebacterium* spp.	1	25	1	10	–	–
	*Bacillus* spp.	–	–	3	30	1	25

*TC* tissue culture (14-day culture of study tissue sample), *SF* sonication fluid (10-day culture of sonication fluid in broth), *MF* membrane-filtrated sonication fluid (cut-off value: ≥ 13.6 CFU/10 ml), *CoNS* coagulase-negative staphylococci, *spp.* species

The diagnostic value of sonication was assessed by comparing the diagnostic performances for the different methods to conventional diagnostics (Table 4). Fourteen-day TC demonstrated high performance. The sensitivity of histopathology was extremely low. SF broth culture had a higher sensitivity than the MF high cut-off, whereas its specificity was lower. In comparison to broth-cultured SF, PPV was higher for the MF high cut-off. Upon using the lower cut-off, the PPV decreased but the NPV was highest of all the SF analysis methods. Regular healers with positive test results were represented across all methods. The MF low cut-off criterion demonstrated the highest positive-test rate in regular healers and the SF broth culture presented the second highest, followed by histopathology. TC and the MF high cut-off had the lowest positive-test rates in regular healers compared to the other methods.

**Table 4:** Performances of different test methods when discriminating between aseptic and septic nonunion

	Septic nonunion	Aseptic nonunion	Sensitivity	Specificity	PPV	NPV	Regular healers
Test method	No. of patients who tested positive		% (95% CI)				No. of patients who tested positive
Long-term TC (study sample)	29/42	2/53	69 (53–82)	96 (86–99)	94 (77–99)	80 (67–88)	3/32
Histopathology (study sample)	6/42	7/53	14 (6–29)	87 (74–94)	46 (20–74)	56 (45–67)	5/32
SF broth culture	29/42	10/53	69 (53–82)	81 (68–90)	74 (58–86)	77 (63–87)	9/32
MF (≥ 0.6 CFU/10 ml)	33/42	20/53	79 (63–89)	62 (48–75)	62 (48–75)	79 (63–89)	10/32
MF (≥ 13.6 CFU/10 ml)	22/42	4/53	52 (37–68)	93 (81–98)	85 (64–95)	71 (59–81)	3/32

*PPV* positive predictive value, *NPV* negative predictive value, *CI* confidence interval, *TC* tissue culture, *SF* sonication fluid, *MF* membrane-filtrated sonication fluid

### Sonication as a confirmatory diagnostic criterion increases sensitivity

To avoid a false-positive infection diagnosis, a confirmatory second criterion should be present. Thus, we analyzed the detection rate of septic nonunion by one positive TC in combination with another positive test method (Table 5). For these analyses, clinical findings were also considered. The same number of patients with septic nonunion could be detected by two TCs with the same pathogen and the combination of TC with SF broth culture. The MF low cut-off as a secondary diagnostic criterion identified the largest number of septic nonunions compared with any other combination.

**Table 5:** Septic nonunion detection rates when using one positive TC in combination with another confirmatory diagnostic criterion

Confirmatory criteria for the diagnosis of septic nonunion	No. of detected septic nonunions	Sensitivity % (95% CI)
≥ 2 Positive TCs with the same pathogen	23/42	55 (39–70)
≥ 1 Positive TC + histopathology with infection signs	10/42	24 (13–40)
≥ 1 Positive TC + same pathogen in SF broth culture	23/42	55 (39–70)
≥ 1 Positive TC + same pathogen in MF (≥ 0.6 CFU/10 ml)	26/42	62 (46–76)
≥ 1 Positive TC + same pathogen in MF (≥ 13.6 CFU/10 ml)	21/42	50 (34–66)

*TC* tissue culture, *CI* confidence interval, *SF* sonication fluid, *MF* membrane-filtrated sonication fluid, *CFU* colony-forming units

## Discussion

Treatment decisions in disturbed fracture healing situations rely on a reliable diagnosis of presence or absence of infection. Established diagnostic criteria for FRI that have been adapted from PJI have so far failed to consider the unique circumstances of fracture healing complications [15, 21]. The central issue in our investigation was to evaluate the diagnostic value of sonication in nonunion differentiation. Our findings suggest that aseptic and septic nonunion can be differentiated by sonication, with sonication being superior to histopathology but inferior to tissue culture. Membrane filtration of sonication fluid in combination with tissue culture increases sensitivity compared to conventional diagnostics and also improves the capacity for detecting polymicrobial infections.

Our study evaluated quantitative CFU thresholds for the diagnosis of FRI, enabling existent infection to be distinguished from contaminated implants [19]. To define the contamination threshold of bacteria usually present on removed implants, we included the group of regular healers. Furthermore, the comparison between septic and aseptic nonunion enabled us to identify a cut-off value for the detection of infection in disturbed fracture healing situations. Both cut-off values demonstrated acceptable discriminatory power [22]. Detection of bacteria in regular healers was most likely due to contamination during surgery or the sampling procedure with skin or airborne microorganisms. Contamination during the laboratory process can be excluded, as negative controls presented no colony growth. Bacterial colonization was higher in aseptic nonunion compared to regular healers. This might be due to bacterial accumulation in non-vital scar tissue in the nonunion zone and colonization of the implant surface. However, this small amount of bacteria did not appear to have any clinical relevance in terms of invading surrounding tissue, as aseptic nonunion with small-scale biofilms on implants did not show further infection signs within 1 year. Bacterial colonization without any clinical relevance was described in patients with aseptic total joint replacement, where 16% demonstrated a positive sonication [23]. Also, bacterial colonization on 40% of routinely removed osteosynthesis material has been reported [2], as it has on 27% of hip implants in patients without infection signs [24]. Such high bacterial colonization rates underline the importance of a CFU cut-off value. Our calculated higher threshold resulted in higher specificity and PPV in comparison to SF broth culture and the lower cut-off. Low microbial loads with no clinical relevance are thus filtered out by the higher cut-off. In contrast, SF in broth leads to bacterial enrichment. 28% of the healers had a positive SF broth culture; using membrane filtration (high cut-off), this number was reduced to 9%.

For sonication, there is no consensus on a uniform sonication protocol or uniform CFU thresholds for infection diagnosis—not even for PJI. Trampuz et al. calculated a CFU cut-off and defined 5 CFU/plate (corresponds to 10 CFU/ml) as the ideal threshold to discriminate between PJI and aseptic failure [19]. However, the CFU cut-offs used in studies range between 1 and 50 CFU/plate [8, 10, 21, 25–30]. Different sonication protocols with variations in the initial fluid volume affect the CFU/ml concentration and prevent the definition of a universal cut-off value. The ROC curves in our study show a relationship between the extent of bacterial implant colonization and the clinical relevance. Thus, we successfully identified SF membrane filtration with CFU quantification as a helpful method to distinguish infected femoral or tibial nonunion from aseptic nonunion or contaminated implants.

However, the value of sonication has to be seen in the context of conventional diagnostics. Therefore, we considered the performances of the diagnostic methods in isolation from each other. With reference to our results, TC shows the highest PPV and NPV. Depending on the SF analysis method or CFU cut-off, sonication can partially match or even exceed TC’s sensitivity, but the specificity of sonication is lower. Onsea et al. reviewed five studies that investigated the accuracy of TC in comparison to sonication. In three studies, sonication demonstrated significantly higher accuracy. However, none of these studies were sufficiently powered, and only one study assessed the diagnostic tests separately for fracture-related infections. Thus, the authors could not conclude that sonication is superior to TC [14]. Our results cannot confirm this either, but they support the value of TC in FRI diagnosis. However, the disadvantages of long-term TC have to be considered. Depending on the clinical standard, long-term culture taking 10–14 days is necessary [1, 31]. In contrast, results from the membrane filtration of SF are available within a few days with relatively high predictive values. This may be beneficial, especially in low-grade infections. These presumably aseptic patients have usually been discharged from the hospital by the time the long-term culture results are available, so antibiotic therapy may not be administered even when clinically indicated. Patients with septic nonunion may also benefit from rapid microbial findings, as their antibiotic therapy can be tailored to the actual bacteria present.

According to our analyses, the method with the poorest sensitivity in the diagnosis of septic nonunion was histopathology, which is in contrast to two previous studies [32, 33]. This may be due to the fact that our study included low-grade infections with osteomyelitis signs that are too weak to be identified with HOES. Egol et al., however, also reported poor sensitivity of frozen (0%) and permanent section histopathology (33%) to infected nonunion [34]. Furthermore, a low sensitivity has been reported in several investigations of the value of histology for differentiating septic and aseptic prosthesis loosening [35–39]. Recently, an international group of experts declared histopathology alone to be a confirmatory criterion for the diagnosis of FRI [15]. With reference to our data, this cannot be confirmed for femur and tibia shaft nonunion. Eight patients with histopathological infection signs as a single criterion were diagnosed as “aseptic,” taking into account the healing course of 1 year. Seven of these patients healed without further infection-specific treatment, while one had not consolidated after 12 months but an underlying infection was excluded. In this context, the positive histopathology test rate of 16% in regular healers should also be mentioned. The value of histopathology in the differential diagnosis of femoral and tibial shaft nonunion should therefore be reconsidered. In view of the good diagnostic performance of the TC shown in our study, more attention should be paid to a single positive long-term TC; for example, if only a few samples were taken in revision surgery because an aseptic nonunion was assumed.

In order to avoid a false-positive FRI diagnosis, the clinical diagnostic standard is based on two criteria, one suggestive and one confirmatory [18]. According to our data for the combination of a TC with the same pathogen in SF, the sensitivity varies depending on the culture method or CFU cut-off. Disagreements about the value of sonication for FRI diagnosis also exist in the current literature. Dudareva et al. investigated the performance of paired TCs and sonication in the diagnosis of PJI and other orthopedic-device-related infections and found that TC was superior [8]. Ueda et al. demonstrated that a more accurate diagnosis of FRI was achieved using a combination of TC or joint aspirate culture with a positive SF culture rather than two positive TCs [21]. Onsea et al. concluded that there is limited evidence of SF being a useful adjunct to TC [14]. With the findings of our study, we have now provided evidence that sonication is a valuable addition to TC: We found that the highest sensitivity for a septic nonunion diagnosis was achieved with a combination of one positive TC and the same pathogen in membrane filtration of SF (low cut-off). Including sonication increases the sensitivity to 62%. The low cut-off is superior to the higher one because five septic nonunions exceed the low cut-off but do not reach the higher threshold. Therefore, we suggest that the lower cut-off should be taken into account to confirm a positive TC.

Nevertheless, based on our results, none of the combined methods demonstrated a sufficient sensitivity. Thus, the diagnosis of septic nonunion should consider different methods to maximize the infection detection rate. For this purpose, sonication seems to be a good additive diagnostic tool that increases sensitivity in combination with TC.

For treatment, it is also important to identify the infection-causing pathogens in order to initiate an appropriate antibiotic therapy. Therefore, we focused on the detected microorganisms. Although culture-negative rates were high in aseptic nonunion and regular healers, the same species present in septic nonunion were still detected in these groups. Common microorganisms in septic nonunion were coagulase-negative staphylococci and *Cutibacterium acnes*. These microorganisms were detected across all methods, which is consistent with other studies [33, 40]. However, the number of species detected in one specimen differed among the different methods. This may be due to the fact that it is more difficult to isolate bacterial strains from culture broth than from a solid culture medium [41]. Additionally, potential competition for nutrients and limited space must be considered in broth cultures [42]. The identification of polymicrobial infections is of clinical relevance due to antibiotic resistances requiring bacteria-specific antibiosis. However, in this context, it should be mentioned that five microorganisms in the SF broth culture could not be detected by membrane filtration of SF. This can be explained not only by different culture media and incubation times but also by the coarse filtration step before membrane filtration which removes small tissue particles, whereas unfiltered SF was inoculated in broth. In two of these cases the same microorganism was identified by TC. Apart from additionally detected species and culture-negative results for individual methods, seven septic nonunions demonstrated discrepant positive culture results between the methods. However, these discrepancies were partly relativized by matching clinical results. Inconsistencies like that and the similar bacteria spectrum across all groups underline that an approach which combines diagnostic methods can be used to avoid false-positive diagnosis.

The study had some limitations. First, SF concentration was achieved by membrane filtration instead of centrifugation. Because membrane filtration has previously shown disadvantages compared to centrifugation [26], we decided to use membrane filtration in a modified form due to the possibility of a higher SF concentration, as we suspected small-scale biofilms of being clinically relevant and negatively affecting fracture healing. Compared to centrifugation, a larger amount of SF could be examined by membrane filtration, corresponding to a microbial examination of 53% of the total implant surface. Regardless, this present study is the first to examine CFU thresholds in FRI, and it was demonstrated that membrane filtration is of value for infection diagnosis.

Second, the incubation period for anaerobic-membrane-filtrated SF cultures was 5 days to obtain countable, distinct colonies, which is short, especially for the detection of *C. acnes*. However, in our study, the anaerobic atmosphere was generated using an Anoxomat system, and evaluation studies of such systems demonstrated that many anaerobic bacteria grow faster under anaerobic conditions created by an Anoxomat than by the conventional gas-pak method [43, 44]. Another study investigated the detection of *C. acnes* growth from orthopedic-implant-associated infections using different culture methods, and detected a growth rate of 99% on sheep blood agar under anaerobic conditions created by an Anoxomat, with a mean and standard deviation of time to detection of 54 ± 10 h [45]. While we cannot rule out the possibility that the short incubation period may have led to an underestimated sensitivity of membrane filtration of sonication fluid with respect to the detection of *C. acnes* in our study, we still assume a high detection rate.

Third, the groups differed in age and fracture type. The regular healer group were younger than the aseptic nonunion group, as fewer healing complications occur in younger patients due to physiological processes and they have fewer open fractures. These differences should not affect the findings from our study, which was on nonunion differential diagnosis.

Furthermore, due to the study design, the number of TC samples differed between septic and aseptic nonunion. Apparently, when the patient demonstrated clinical infection signs, surgeons collected more samples to identify the infection-causing bacteria. Despite this, we are confident that all septic and aseptic nonunions were accurately identified, as we were able to follow up the healing course for 1 year to get the definitive diagnosis.

Finally, it should be noted that TC is the gold standard in FRI diagnostics and was also decisive for septic nonunion identification in the present study. Therefore, evaluating the diagnostic performance of TC is difficult. Nevertheless, in order to compare TC with other methods, we only considered the additional study TC that was processed in a standardized manner for performance analyses.

These limitations are countered by significant strengths of the study. In contrast to other studies that processed samples from multiple sites [2, 8–13, 21, 25–27, 29] or included prosthetic infections [8, 9, 11–13, 21, 26], the present study included only osteosynthesis implants of femur and tibia shafts. Furthermore, in contrast to other studies [2, 8–10, 23, 25–29, 46], the entire implants were sonicated, which makes sonication results more comparable between patients and improves the reliability of CFU thresholds. Finally, a great strength is the inclusion of a group of regular healers. Thus, it was possible to take bacterial contamination of removed implants into account.

In conclusion, the results of this present study support a multimodal approach for the differential diagnosis of infection in fracture nonunion, including clinical, microbiological and histopathological findings. Among these different methods, sonication was demonstrated to have substantial usefulness. Membrane filtration of sonication fluid improves the differential diagnosis in nonunion, reduces the time to culture positivity, and detects polymicrobial infections more frequently. These are important clinical aspects for an early initiation of bacteria-specific antibiosis.

## Data Availability

The datasets generated and analyzed during the current study are available from the corresponding author on reasonable request.

## Abbreviations

AUC: Area under the curve
CFU: Colony-forming units
CI: Confidence interval
CoNS: Coagulase-negative staphylococci
FRI: Fracture-related infection
HOES: Histopathological Osteomyelitis Evaluation Score
MF: Membrane filtration
NPV: Negative predictive value
PJI: Prosthetic joint infection
PPV: Positive predictive value
ROC: Receiver operating characteristic
SF: Sonication fluid
TC: Tissue culture

## References

[1] Hackl S, Keppler L, von Rüden C, Friederichs J, Perl M, Hierholzer C (2021). The role of low-grade infection in the pathogenesis of apparently aseptic tibial shaft nonunion. Injury.

[2] Dapunt U, Spranger O, Gantz S, Burckhardt I, Zimmermann S, Schmidmaier G (2015). Are atrophic long-bone nonunions associated with low-grade infections?. Ther Clin Risk Manag.

[3] Nelson CL, McLaren AC, McLaren SG, Johnson JW, Smeltzer MS (2005). Is aseptic loosening truly aseptic?. Clin Orthop Relat Res.

[4] Neut D, van Horn JR, van Kooten TG, van der Mei HC, Busscher HJ (2003). Detection of biomaterial-associated infections in orthopaedic joint implants. Clin Orthop Relat Res.

[5] Oliva A, Miele MC, Al Ismail D, Di Timoteo F, De Angelis M, Rosa L (2021). Challenges in the microbiological diagnosis of implant-associated infections: a summary of the current knowledge. Front Microbiol.

[6] Van Diek FM, Albers CGM, Van Hooff ML, Meis JF, Goosen JHM (2017). Low sensitivity of implant sonication when screening for infection in revision surgery. Acta Orthop.

[7] Grosso MJ, Frangiamore SJ, Yakubek G, Bauer TW, Iannotti JP, Ricchetti ET (2018). Performance of implant sonication culture for the diagnosis of periprosthetic shoulder infection. J Shoulder Elbow Surg.

[8] Dudareva M, Barrett L, Figtree M, Scarborough M, Watanabe M, Newnham R (2018). Sonication versus tissue sampling for diagnosis of prosthetic joint and other orthopedic device-related infections. J Clin Microbiol.

[9] Puig-Verdié L, Alentorn-Geli E, González-Cuevas A, Sorlí L, Salvadó M, Alier A (2013). Implant sonication increases the diagnostic accuracy of infection in patients with delayed, but not early, orthopaedic implant failure. Bone Jt J.

[10] Yano MH, Klautau GB, da Silva CB, Nigro S, Avanzi O, Mercadante MT (2014). Improved diagnosis of infection associated with osteosynthesis by use of sonication of fracture fixation implants. J Clin Microbiol.

[11] Portillo ME, Salvadó M, Trampuz A, Siverio A, Alier A, Sorli L (2015). Improved diagnosis of orthopedic implant-associated infection by inoculation of sonication fluid into blood culture bottles. J Clin Microbiol.

[12] Esteban J, Gomez-Barrena E, Cordero J, Martín-de-Hijas NZ, Kinnari TJ, Fernandez-Roblas R (2008). Evaluation of quantitative analysis of cultures from sonicated retrieved orthopedic implants in diagnosis of orthopedic infection. J Clin Microbiol.

[13] Holinka J, Bauer L, Hirschl AM, Graninger W, Windhager R, Presterl E (2011). Sonication cultures of explanted components as an add-on test to routinely conducted microbiological diagnostics improve pathogen detection. J Orthop Res Off Publ Orthop Res Soc.

[14] Onsea J, Depypere M, Govaert G, Kuehl R, Vandendriessche T, Morgenstern M (2018). Accuracy of tissue and sonication fluid sampling for the diagnosis of fracture-related infection: a systematic review and critical appraisal. J Bone Jt Infect.

[15] Govaert GAM, Kuehl R, Atkins BL, Trampuz A, Morgenstern M, Obremskey WT (2020). Diagnosing fracture-related infection: current concepts and recommendations. J Orthop Trauma.

[16] Fisher JS, Kazam JJ, Fufa D, Bartolotta RJ (2019). Radiologic evaluation of fracture healing. Skeletal Radiol.

[17] Schmidmaier G, Moghaddam A (2015) Long bone nonunion. Z Orthop Unfall. 10.1055/s-0035-1558259 10.1055/s-0035-1558259 26670151

[18] Metsemakers WJ, Morgenstern M, McNally MA, Moriarty TF, McFadyen I, Scarborough M (2018). Fracture-related infection: a consensus on definition from an international expert group. Injury.

[19] Trampuz A, Piper KE, Jacobson MJ, Hanssen AD, Unni KK, Osmon DR (2007). Sonication of removed hip and knee prostheses for diagnosis of infection. N Engl J Med.

[20] Tiemann A, Hofmann GO, Krukemeyer MG, Krenn V, Langwald S (2014). Histopathological osteomyelitis evaluation score (HOES)—an innovative approach to histopathological diagnostics and scoring of osteomyelitis. GMS Interdiscip Plastic Reconstr Surg DGPW.

[21] Ueda N, Oe K, Nakamura T, Tsuta K, Iida H, Saito T (2019). Sonication of extracted implants improves microbial detection in patients with orthopedic implant-associated infections. J Arthroplasty.

[22] Hosmer DW, Lameshow S, Sturdivant RX (2013) Assessing the fit of the model. In: Hosmer DW, Lemeshow S, Sturdivant RX (eds) Applied logistic regression, 3rd edn. Wiley, New York

[23] Rothenberg AC, Wilson AE, Hayes JP, O'Malley MJ, Klatt BA (2017). Sonication of arthroplasty implants improves accuracy of periprosthetic joint infection cultures. Clin Orthop Relat Res.

[24] Fuchs M, Kinzel S, Gwinner C, Perka C, Renz N, von Roth P (2019) Clinically asymptomatic patients show a high bacterial colonization rate of osteosynthetic implants around the knee but not the hip. J Arthroplasty 34:1761–1766. 10.1016/j.arth.2019.03.058 10.1016/j.arth.2019.03.058 31064723

[25] Maniar HH, Wingert N, McPhillips K, Foltzer M, Graham J, Bowen TR (2016). Role of sonication for detection of infection in explanted orthopaedic trauma implants. J Orthop Trauma.

[26] Zitron R, Wajsfeld T, Klautau GB, da Silva CB, Nigro S, Mercadante MT (2016). Concentration of sonication fluid through centrifugation is superior to membrane filtration for microbial diagnosis of orthopedic implant-associated infection. J Clin Microbiol.

[27] Portillo ME, Salvadó M, Trampuz A, Plasencia V, Rodriguez-Villasante M, Sorli L (2013). Sonication versus vortexing of implants for diagnosis of prosthetic joint infection. J Clin Microbiol.

[28] Cazanave C, Greenwood-Quaintance KE, Hanssen AD, Karau MJ, Schmidt SM, Gomez Urena EO (2013). Rapid molecular microbiologic diagnosis of prosthetic joint infection. J Clin Microbiol.

[29] Achermann Y, Vogt M, Leunig M, Wüst J, Trampuz A (2010). Improved diagnosis of periprosthetic joint infection by multiplex PCR of sonication fluid from removed implants. J Clin Microbiol.

[30] Piper KE, Jacobson MJ, Cofield RH, Sperling JW, Sanchez-Sotelo J, Osmon DR (2009). Microbiologic diagnosis of prosthetic shoulder infection by use of implant sonication. J Clin Microbiol.

[31] Schäfer P, Fink B, Sandow D, Margull A, Berger I, Frommelt L (2008). Prolonged bacterial culture to identify late periprosthetic joint infection: a promising strategy. Clin Infect Dis.

[32] Simpson AH, Wood MK, Athanasou NA (2002). Histological assessment of the presence or absence of infection in fracture non-union. Injury.

[33] Morgenstern M, Athanasou NA, Ferguson JY, Metsemakers WJ, Atkins BL, McNally MA (2018). The value of quantitative histology in the diagnosis of fracture-related infection. Bone Jt J.

[34] Egol KA, Karunakar MA, Marroum MC, Sims SH, Kellam JF, Bosse MJ (2002). Detection of indolent infection at the time of revision fracture surgery. J Trauma.

[35] Bori G, Soriano A, García S, Gallart X, Casanova L, Mallofre C (2006). Low sensitivity of histology to predict the presence of microorganisms in suspected aseptic loosening of a joint prosthesis. Mod Pathol.

[36] Abdul-Karim FW, McGinnis MG, Kraay M, Emancipator SN, Goldberg V (1998). Frozen section biopsy assessment for the presence of polymorphonuclear leukocytes in patients undergoing revision of arthroplasties. Mod Pathol.

[37] Ko PS, Ip D, Chow KP, Cheung F, Lee OB, Lam JJ (2005). The role of intraoperative frozen section in decision making in revision hip and knee arthroplasties in a local community hospital. J Arthroplasty.

[38] Banit DM, Kaufer H, Hartford JM (2002). Intraoperative frozen section analysis in revision total joint arthroplasty. Clin Orthop Relat Res.

[39] Musso AD, Mohanty K, Spencer-Jones R (2003). Role of frozen section histology in diagnosis of infection during revision arthroplasty. Postgrad Med J.

[40] Otchwemah R, Moczko T, Marche B, Mattner F, Probst C, Tjardes T (2020) High prevalence of bacteria in clinically aseptic non-unions of the tibia and the femur in tissue biopsies. Eur J Trauma Emerg Surg 46:1093–1097. 10.1007/s00068-018-1010-z 10.1007/s00068-018-1010-z 30255295

[41] Bonnet M, Lagier JC, Raoult D, Khelaifia S (2020). Bacterial culture through selective and non-selective conditions: the evolution of culture media in clinical microbiology. New Microbes New Infect.

[42] Hibbing ME, Fuqua C, Parsek MR, Peterson SB (2010). Bacterial competition: surviving and thriving in the microbial jungle. Nat Rev Microbiol.

[43] Shahin M, Jamal W, Verghese T, Rotimi VO (2003). Comparative evaluation of anoxomat and conventional anaerobic GasPak jar systems for the isolation of anaerobic bacteria. Med Princ Pract.

[44] Summanen PH, McTeague M, Väisänen ML, Strong CA, Finegold SM (1999). Comparison of recovery of anaerobic bacteria using the anoxomat, anaerobic chamber, and GasPak jar systems. Anaerobe.

[45] Jeverica S, El Sayed F, Čamernik P, Kocjančič B, Sluga B, Rottman M (2020). Growth detection of cutibacterium acnes from orthopaedic implant-associated infections in anaerobic bottles from BACTEC and BacT/ALERT blood culture systems and comparison with conventional culture media. Anaerobe.

[46] Evangelopoulos DS, Stathopoulos IP, Morassi GP, Koufos S, Albarni A, Karampinas PK (2013). Sonication: a valuable technique for diagnosis and treatment of periprosthetic joint infections. Sci World J.

